# High frequency abrupt shifts in the Indian summer monsoon since Younger Dryas in the Himalaya

**DOI:** 10.1038/s41598-018-27597-6

**Published:** 2018-06-18

**Authors:** Sheikh Nawaz Ali, Jyotsna Dubey, Ruby Ghosh, M Firoze Quamar, Anupam Sharma, P Morthekai, A P Dimri, Mayank Shekhar, Md. Arif, Shailesh Agrawal

**Affiliations:** 1 Birbal Sahni Institute of Palaeosciences, Lucknow, India; 2 School of Environmental Sciences, Jawaharlal Nehru University, New Delhi, India

## Abstract

In order to quantify the Indian summer monsoon (ISM) variability for a monsoon dominated agrarian based Indian socio-economy, we used combined high resolution δ^13^C, total organic carbon (TOC), sediment texture and environmental magnetic data of the samples from a ~3 m deep glacial outwash sedimentary profile from the Sikkim Himalaya. Our decadal to centennial scale records identified five positive and three negative excursions of the ISM since last ~13 ka. The most prominent abrupt negative ISM shift was observed during the termination of the Younger Dryas (YD) between ~11.7 and 11.4 ka. While, ISM was stable between ~11 and 6 ka, and declined prominently between 6 and 3 ka. Surprisingly, during both the Medieval Warm Period (MWP) and Little Ice age (LIA) spans, ISM was strong in this part of the Himalaya. These regional changes in ISM were coupled to southward shifting in mean position of the Intertropical Convergence Zone (ITCZ) and variations in East Asian monsoon (EAM). Our rainfall reconstructions are broadly in agreement with local, regional reconstructions and PMIP3, CSIRO-MK3L model simulations.

## Introduction

**Figure 1:**
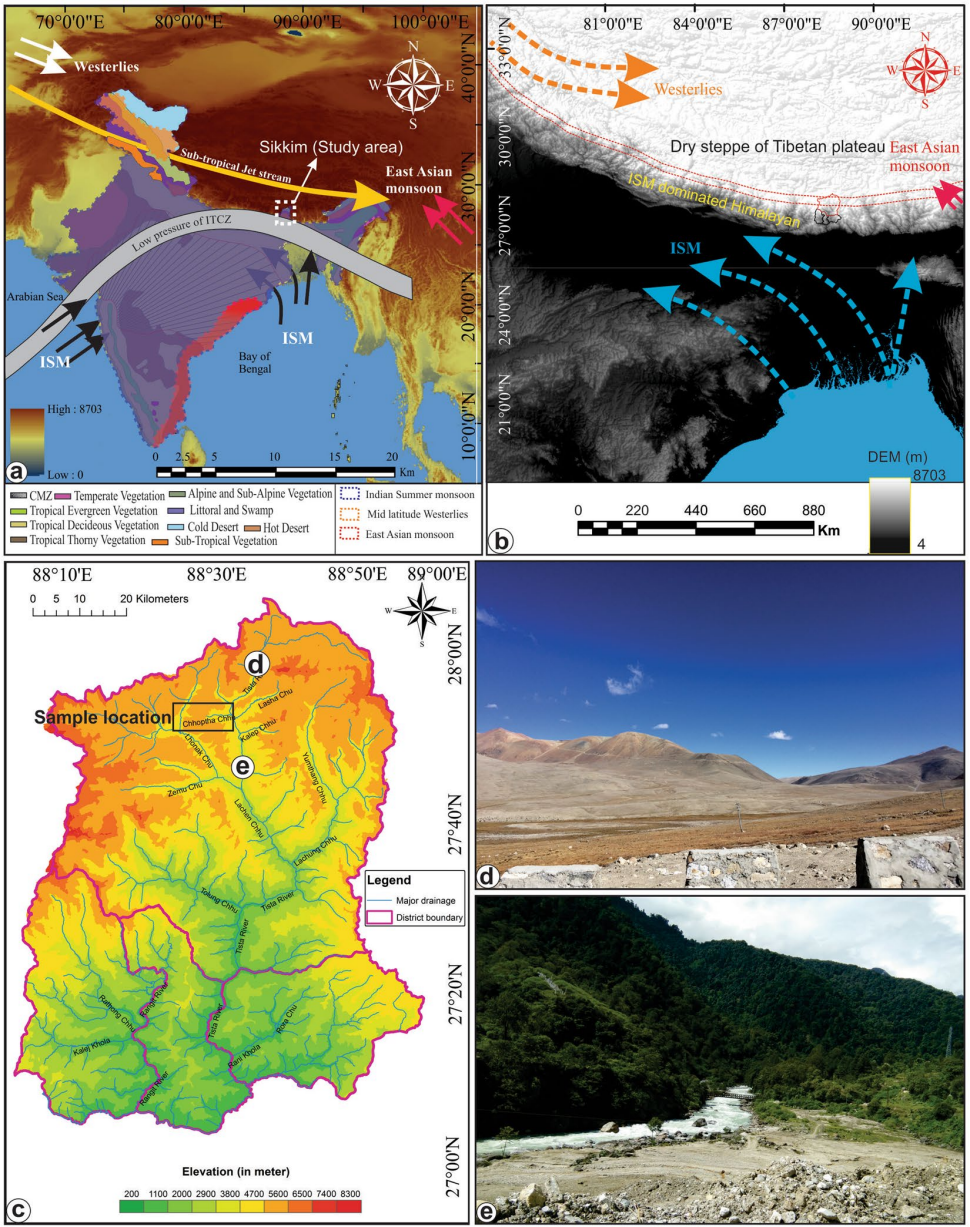
Map compilation showing (**a**) the location of the study area i.e. the Sikkim Himalaya in India with different vegetation zones and weather systems (modified after Dixit & Tandon^71^ and Zorzi *et al*.^72^); (**b**) shuttle radar topographic mission (SRTM) digital elevation model (DEM) of central and eastern parts of India showing the study area, Sikkim (outlined in red) and the approximate transitional zone between the dry steppe of the trans-Himalaya towards the north and the sub-humid Himalaya to the south (marked between two red dashed lines); (**c**) Digital elevation model showing the major drainage and the location of sampling sites; (**d**) Dry steppe vegetation towards the north of the sampling sites. Location of the photographs is shown in Fig. 1c. (The maps are created using ArcGIS Version 10.2) and (**e**) Field photograph showing sub humid vegetation towards south of sampling sites.

**Figure 2:**
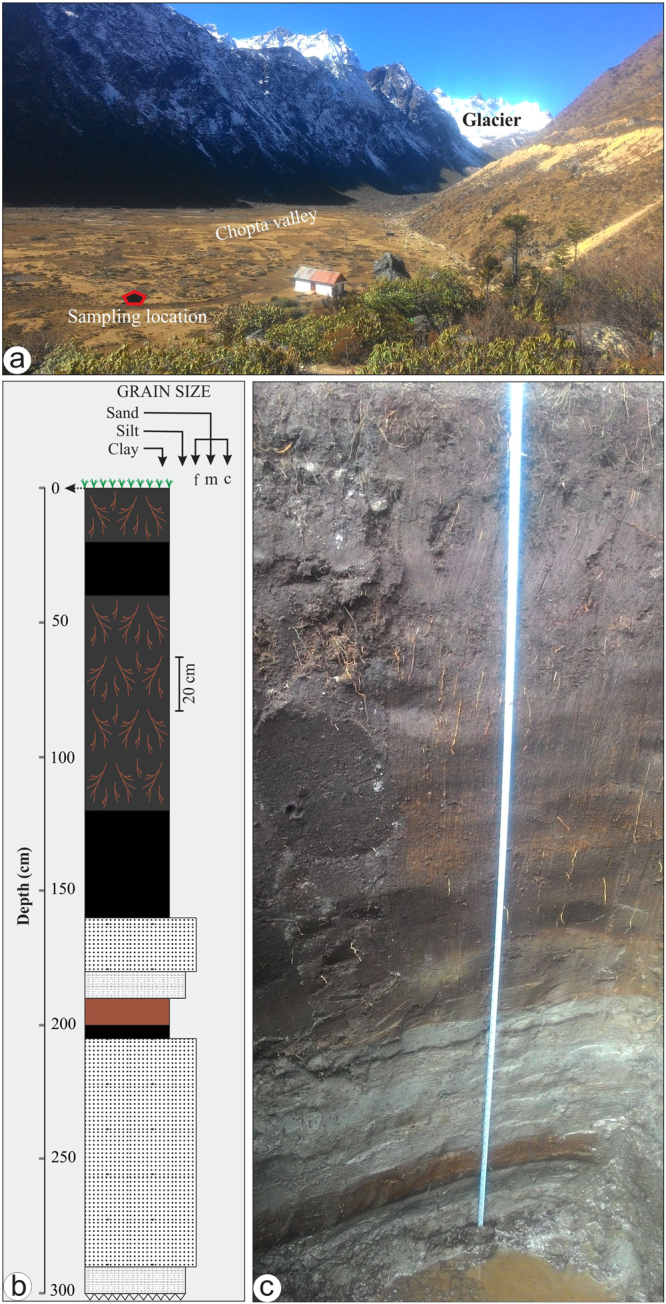
(**a**) Field photograph taken facing upstream direction, showing the synoptic view of the Chopta valley. The red pentagon shows the sampling location. (**b**) Detailed litholog of the sedimentary profile. (**c**) Field photograph showing the texture of the sedimentary profile.

**Figure 3:**
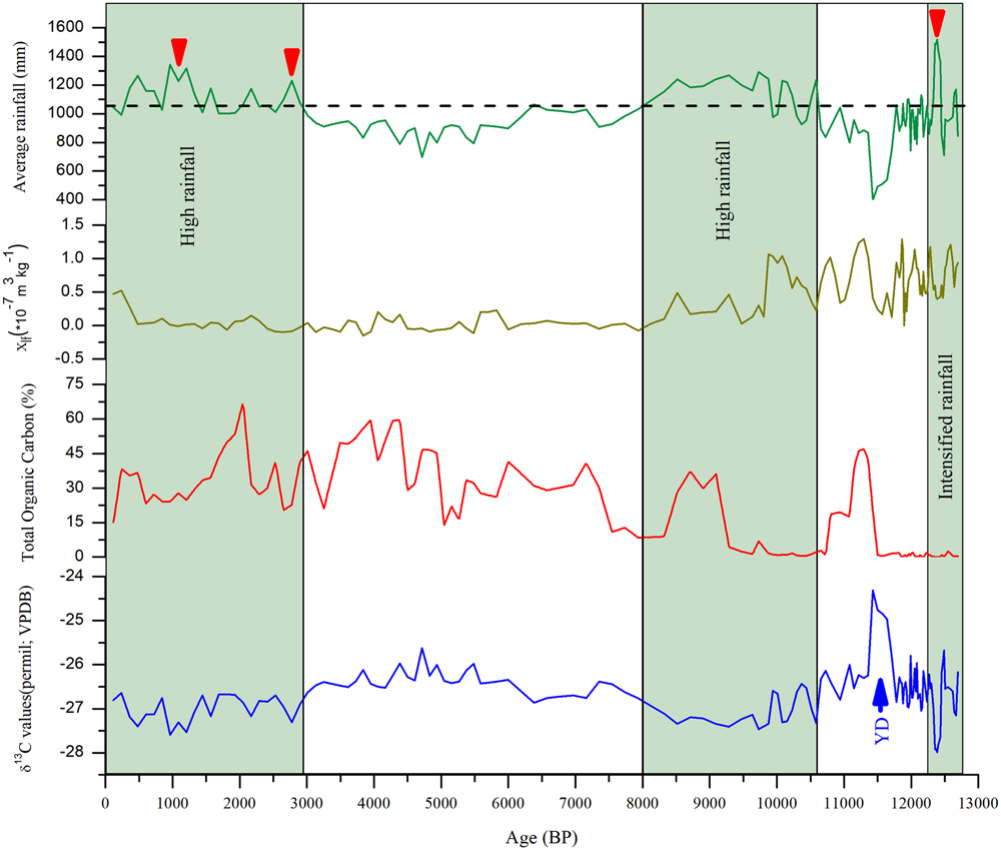
Measured δ^13^C value (‰; VBDB; blue line), total organic carbon (TOC; %; red line) and low field magnetic susceptibility (10^-7^m^3^kg^-1^; olive green line) of the sedimentary profile are shown against the depositional age (BP; ^14^C AMS). The top green line shows the average reconstructed palaeoprecipitation values (mm) based on the δ^13^C values using emperical relationship given in Kohn^38^ and Basu *et al.*^39^.

Indian summer monsoon (ISM) is a vital source of precipitation and significant component of Asian monsoon system and global climate. It originates by seasonal reversal of winds due to differential heating of the Indian subcontinent and Tibetan Plateau^1–3^. By contributing over 80% of the total annual rainfall in India^4^, ISM significantly influences agrarian based Indian socio-economy and is considered as the most significant weather system nourishing the Himalayan glaciers^5–7^. Abrupt shifts in the ISM on decadal to centennial scales might severely affect Indian socio-economy as observed in the recent past^8^ and is associated with the rise and fall of ancient human civilisations^9–12^. In order to improve the predictive capabilities of the future ISM variability, characterization of the ISM precipitation before the instrumental period is becoming an urgent need. To understand the trends in the ISM variability, high resolution proxy records of precipitation with greater temporal and geographic coverage are necessary. Most of the records of the ISM variability during the Late Pleistocene to Holocene, when earth experienced several abrupt climatic shifts, are generated from marine archives primarily from Arabian Sea^13–19^ and Bay of Bengal^20,21^ while, from terrestrial archives high resolution data is scanty^11,22–31^ and is often suffering from age uncertainties. Therefore, ISM variability should be explored with a terrestrial high resolution monsoon proxy record with a greater temporal coverage to assess the inferred role of solar forcing^16–18^, teleconnections to North Atlantic climate changes^13,16^ and variations in the East Asian monsoon (EAM)^21^ prior to the last glacial and Holocene. The Himalaya, an underexplored yet significant region acting as one of the ‘modulators of the ISM’ together with the Tibetan Plateau (TP), is the better, if not ideal region for quantifying past ISM variability. Although, some work has been done in the eastern Indian Hiamalaya, however, most of the records are qualitative and showed incongruity (Fig. S1). The climatologically sensitive transitional zone lying between the dry Trans Himalaya/Tibet towards north and the sub-humid Himalaya towards south that receives high precipitation (amplified signal) during intensified ISM and vice-versa is most suitable in this regard (Fig. 1). Our study area, the Chopta valley, a proglacial outwash plain in north Sikkim Himalaya fulfills the erstwhile conditions (Figs 1 and 2). Here we present a high resolution carbon isotope signature (**δ**^13^C) preserved in sediment organic matter (SOM) of Chopta valley combined with total organic carbon (TOC), sediment texture and environmental magnetic data to document the ISM variability in the last ~13 ka (Fig. 3), which also helped unraveling the forcing factors responsible for ISM variability. To explore the link between ISM variability with northern high latitude climate changes during the last deglaciation, we have compared our data with regional marine, speleothem and global ice core records. The model simulation outputs are giving significantly comparable results. The model study is also performed to see spatial extent of ISM during past climate change scenario.

### δ^13^C and Total Organic Carbon (TOC) content in SOM and bulk sediment κ_lf_

The **δ**^13^C values in the SOM are significantly used as a climate proxy for regional precipitation given that the organic matter source is C_3_ plants^32–36^ (see supplimentary data for details). From the variations observed in TOC content of the profile, it is observed that the detrital input (glacial sediments) and environmental factors have significantly affected the TOC content of the profile^35^. The lower half of the sediment profile (~300 to 160 cm; ~12 to 10 ka) having coarse lithology and high sedimentation rate show very low TOC with a couple of high excurtions. However, the upper half of the profile (~160 to top; 10 ka to present) has high TOC suggesting a homogenised and favorable environment for floral growth and organic matter preservation (Figs 2 and 3).

To understand the isotopic response of the vegetation towards rainfall, we have generated a modern analogue using SOM of surface samples. The **δ**^13^C values of SOM of the Chopta valley vary between −25.8 and −27.7‰ (average −26.6‰) showing little variation and suggesting an overall dominance of C_3_ plants. Moreover, it has also been observed that the **δ**^13^C values do not exhibit any significant trend between the hill slope and valley floor samples suggesting an isotopically homogenized system^37^. The **δ**^13^C values used to quantifty the present day precipitation using the equations proposed by Kohn *et al*.^38^ and Basu *et al*.^39^ are in ageement and verified with CRU TS 3.24 data^40^. The **δ**^13^C values of de-carbonated samples in a ~3 m deep sedimentary profile vary between −28.0 and −24.3‰ having a significant difference of 3.7‰ (mean value −26.7‰) (Fig. 3; Table S1). Chronology of deposition for the Chopta valley profile is developed using a Bayesian age model using five ^14^C AMS radiocarbon dates. The magnetic susceptibility (MS) shows a definite fluctuating trend reflecting lithological changes throughout the profile. The sandy horizons show maximum MS values while the organic peaty layers have minimum MS values (Supplementary Information 1).

### Palaeo-precipitation reconstruction and correlation

Unlike the western and central Himalaya, dominated by extra-tropical westerlies during the winters, the Eastern Himalaya is affected by the thermal gradient between the Bay of Bengal and Indian subcontinent^41,42^ and is dominated by the ISM precipitation. Although the amount of rainfall keeps on decreasing from south to north across the Himalaya, however, the transitional zones receive good amount of rainfall during intensified ISM, making them ideal place to study ISM variability.

Here we present a quantitative data for the ISM variability deduced through carbon isotopic data of SOM following Kohn *et al*.^38^ and Basu *et al*.^39^ (Fig. 3) in the last ~12.7 ka from a sediment profile mentioned above (Figs 2 and 3). It is observed that during the Pleistocene−Holocene transition, the ISM witnessed abrupt high frequency fluctuations^16–19,21^. Our combined data show that during the entire span of the Younger Dryas (YD) stadial between ~12.7 and 11.6 ka, the ISM was weaker than now, but around 12.4 ka an abrupt positive centennial scale excursion (~42% increase in rainfall) has been noticed, possibly the most wettest one within the last deglacial period. The most pronounced and abrupt weakening (~63% drop in rainfall) in the ISM has been observed at the termination of the YD between ~11.7 and 11.4 ka. Subsequently, a gradual rising trend for the ISM is noticed until ~10.6 ka, but still with lower rainfall than present. The overall weak ISM between ~12.7 and 11.6 ka may be attributed to the weakening of the Atlantic Meridional Overturning Circulation (MOC) that occurred during the YD^41^. Furthermore, central and eastern Himalayan glaciers showed a short term advancement during this period, that has been attributed to lower temperature^42–44^. Being in the domain of ISM we believe that the glaciers advanced due to lower temperatures during the YD that sustained and nourished by the wet early Holocene. Similar weakening in the ISM during the YD is observed by other workers also^18,19,21,44–47^. Our estimation shows that the rainfall dropped to a minimum of ~400 mm at the termination of the YD with a deficit of ~650 mm from normal. The YD, characterized by weak ISM and strengthened westerlies^22,48,49^, is a canonical abrupt climatic event that has been linked to marked reduction in the North Atlantic meridional overturning circulation (MOC), and a shutdown or weakening of the MOC^41,50–52^. Further, some recent records inferred that ISM precipitation was linked to variations in both the North Atlantic climate and EAM on multicentennial to millennial time scales^18,22,53^. The increase in latitudinal thermal gradient drove stronger westerly winds during the YD cold span, and southward shift in mean position of ITCZ led to weakening of both the ISM and EAM^53^. It is assumed that there is little influence of Western disturbances (WD) or westerly’s on the Eastern Himalaya, however, a centennial scale abrupt strengthening in ISM between ~12.4 and 12.3 ka within the YD is surprising (Fig. 3). This abrupt strengthening in ISM might be due to interaction of WDs with a break in the summer monsoon trough which might have led to heavy rainfall for a brief spell in the Eastern Himalaya^54^.

Gradual strengthening of the ISM after the YD glacial cooling event is recorded and is punctuated by two centennial to millennial scale precipitation minima phases during ~10.6-8.0 ka. It is observed that during this phase an increase of up to ~21% in ISM precipitation is witnessed. Recently, Bhushan *et al*.^55^ also inferred a similar gradual strengthening of ISM after the YD from monsoon dominated central Himalaya using detrital proxies. Post YD strengthening of ISM has been documented in both marine and terrestrial proxies on regional scale^14,15,19,20,22,45,48,56–61^ (Fig. S3). The early Holocene ISM intensification has been attributed to two inherently related different processes namely boreal summer insolation considered as the external forcing mechanism^15,62^ and significant reduction in albedo due to decrease in Tibetan Plateau snow cover^63^. The strong and stable ISM (~10.6-8.0 ka) is followed by a gradual decline during ~8 to 3 ka and is punctuated by a moderate ISM event recorded at ~6.4 ka (Fig. 2). It is observed that there has been a slight fluctuation in the precipitation between ~8 and 6 ka and is followed by a gradual decrease in precipitation that persisted until ~3 ka.

Our observations broadly corroborate with the inferences of a moderate surface runof f ^55^ from a similar ISM dominated region from the Central Himalaya. The gradual fluctuating trend seen in the ISM precipitation post ~8 ka is also accorded by coupled atmosphere-ocean parameters used in global climate model simulations^64^. Further, our data is also in correspondence with that of ocean data sets suggesting similar ocean-atmospheric conditions relative to present and matches well even with the mid-Holocene period (ca. 6000 yrs BP)^65,66^. The fluctuating decreasing trend in the rainfall pattern post ~6 ka is attributed to the progressive decrease in insolation and south ward shift of the ITCZ after mid Holocene. Stabilization of ISM took place after ~3 ka and is broadly punctuated by three enhanced ISM events at ~2.8 ka (15%), 2.1 ka (10%) and during 1300 – 400 (26%) yr BP. The sharp fluctuating trend with rainfall reaching to its maximum (~26% above normal) during ~1300 to 400 yrs BP corresponding to Medieval warm period (MWP), an intensified and wet ISM phase^40,67^. It is also inferred that after ~300 yrs BP (Little Ice age) amelioration in the ISM is recorded and corroborates well with tree ring based records of Shekhar *et al*.^68^. Surprisingly our data show no drop in ISM precipitation during the LIA in the Sikkim Himalaya attributable to the dual interactions of ITCZ and strong WDs^61,69^ during this phase. A high frequency of monsoon break events during ~1400 and 1700 AD might have cumulatively generated centennial-scale episodes of negative precipitation anomalies over central India and positive precipitation anomalies over northeast India^70^. Sinha *et al*.^70^ inferred influence of planetary-scale ITCZ changes on ISM precipitation by modulating the frequency of active-break periods which is further corroborated by our results.

**Figure 4:**
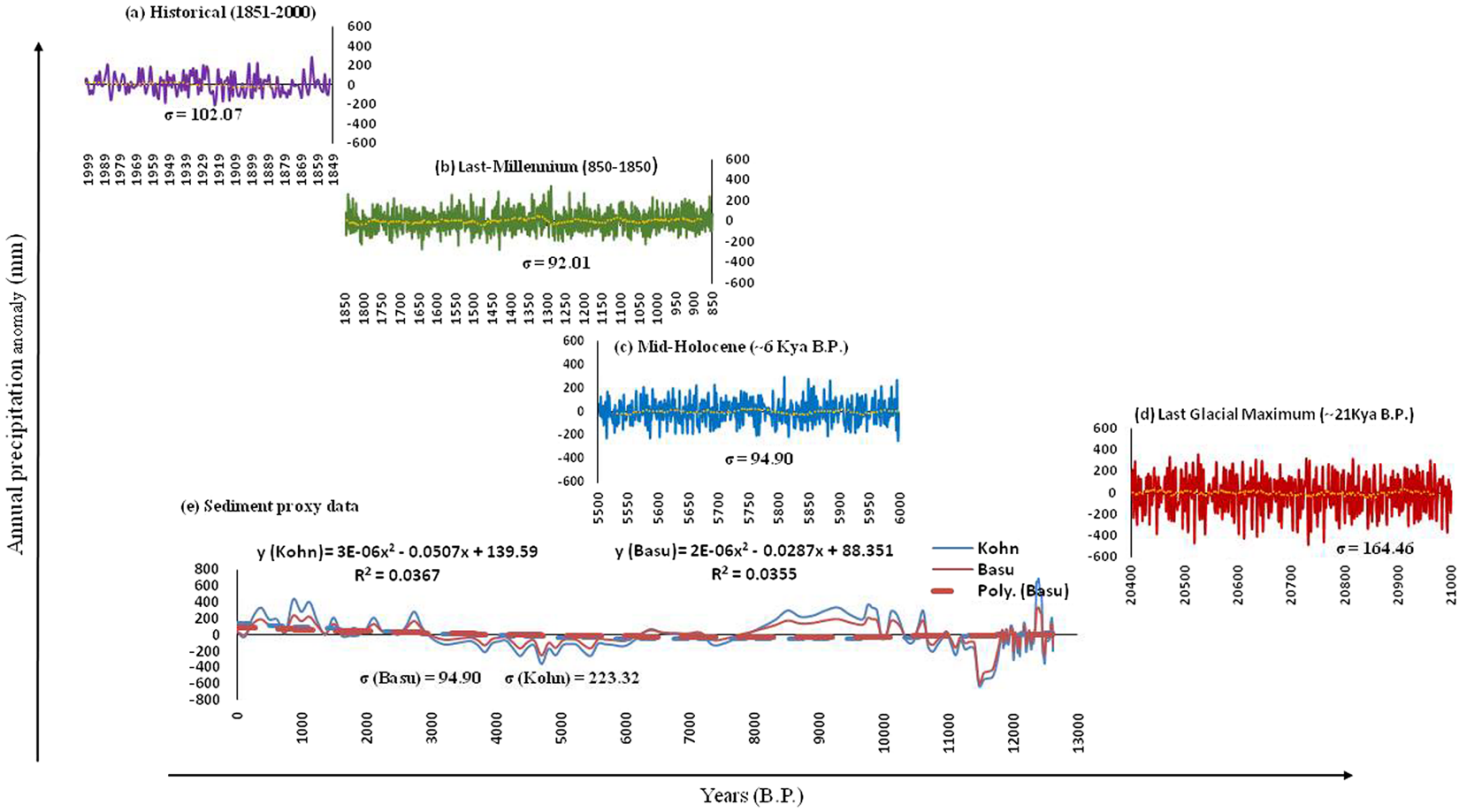
(**a**) Variations of annual rainfall anomaly (mm) averaged over core monsoon region (73–82°E; 18–28°N) during (**a**) Historical (1850–2005), (**b**) Last Millennium (850–1849), (**c**) Mid-Holocene (~6 kya before present (B.P.) and (**d**) Last Glacial Maximum (LGM; ~21 kya B.P.) over Indian region from IPSL-CM5A-LR for (**a**), CSIRO-Mk3L for (**b**) and (**c**) and COSMOS-ASO for (**d**) global climate models respectively. For Mid-Holocene and Last glacial maximum variations are shown over a period of 500 and 600 years respectively. For each period, ‘σ’ represents the standard deviation over the years while the line curves show the 33 years moving average over that period. (**e**) Precipitation record derived from proxy sediment core data from two locations in the Upper Sikkim valley where dashed line in corresponding colors with y-x equation represents the second degree polynomial regression fit. The inter-annual variability of rainfall gradually decreased from last glacial maximum to historical as is clearly evident from ‘σ’ value.

**Figure 5:**
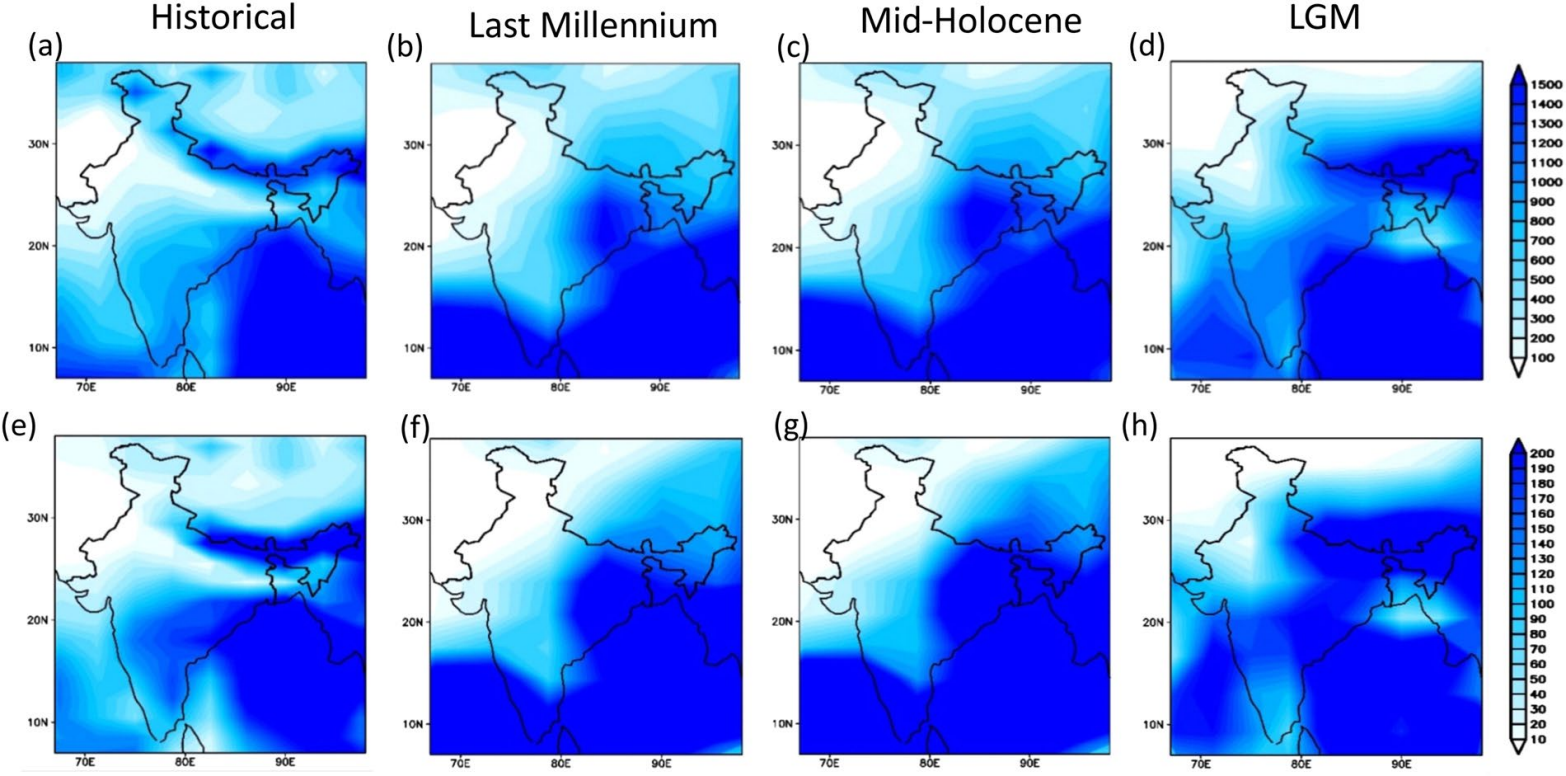
Variations of Annual precipitation climatology (mm) (**a**–**d**) and JJAS precipitation climatology (mm/month) (**e**–**h**) over Indian sub-continental region (67–98°E; 7–38°N) during (**a**,**e**) Historical (1850–2005), (**b**,**f**) Last Millennium (850–1849), (**c**,**g**) Mid-Holocene (~6 kya B.P.) and (**d**,**h**) Last Glacial Maximum (LGM; ~21 kya B.P.) from IPSL-CM5A-LR for (**a**,**e**), CSIRO-Mk3L for (**b**,**f**) and (**c**,**g**) and COSMOS-ASO for (**d**,**h**) global climate models respectively. For Mid-Holocene and Last glacial maximum variations are shown over a period of 500 and 600 years respectively.

Our **δ**^13^C based palaeoprecipitation reconstruction from the eastern Himalaya is in great agreement with the regional terrestrial and marine palaeorecords as well as with those of glacial reconstructions from the adjoining areas. It is evident from our data that during the Pleistocene-Holocene boundary the ISM was very unstable. The broad correlation between different proxy data sets suggests common forcing factors that need to be further understood by generating high resolution palaeoprecipitation data sets from ecologically diverse climatic regions of the Indian subcontinent (Fig. S3). We show that the early phase of the Holocene has witnessed dramatic centennial to millennial scale abrupt precipitation fluctuations. Although, qualitative reconstructions suggest a marginally weak to moderate ISM during the mid-Holocene, however, it is now apparent from our study that the precipitation was marginally low with respect to present and punctuated by centennial scale dry spells (Fig. 3). Further, it is suggested that the area seems to have been under the influence of enhanced mid-latitude westerlies during the dry spells like YD and LIA. The model output shows gradual change in precipitation pattern from Last Glacial Maximum (LGM) to Historical (Figs 4 and 5). The variations in precipitation among LGM, mid Holocene and Historical periods are also clearly seen. As the model shows precipitation pattern shift over eastern to western Himalaya from mid Holocene to Historical period, that also delineates the reconstruction of precipitation over western Himalaya after Indus valley civilization ruin.

## Electronic supplementary material

Supplementary Information
