# Supplementary material for: High frequency abrupt shifts in the Indian summer monsoon since Younger Dryas in the Himalaya
Source: Sci Rep. 2018 Jun 18;8:9287. doi: 10.1038/s41598-018-27597-6 (PMC6006316; doi:10.1038/s41598-018-27597-6)
Supplement: Supplementary file 1 — Supplementary Information [file 41598_2018_27597_MOESM1_ESM.pdf]

High frequency abrupt shifts in the Indian summer monsoon since Younger Dryas in the Himalaya

Sheikh Nawaz Ali[1]*, Jyotsna Dubey[1], Ruby Ghosh[1], M Firoze Quamar[1], Anupam Sharma[1], P Morthekai[1], A P Dimri[2], Mayank Shekhar[1], Md. Arif[1] and Shailesh Agrawal[1]*

1. Birbal Sahni Institute of Palaeosciences, Lucknow, India

2. Jawaharlal Nehru University (JNU), New Delhi, India

*snawazali@gmail.com; as.shail@gmail.com

Supplementary

(1) Stable carbon isotopes and palaeoclimate: an introduction

The $\delta^{13}C$ value of C_3 plants is controlled by the fractionation associated with atmospheric carbon dioxide (CO_2) uptake and fixation (Δ) during photosynthesis and can be described by mathematical model using the formula ($\Delta = a + (b - a)(p_i/p_a)$)[1]. Here, ‘a’ and ‘b’ are the constant values of isotopic fractionation during diffusion of CO_2 through stomata (4.4‰) and during carboxylation (~29‰), respectively. 'p_i' and 'p_a' are the intercellular and ambient partial pressures of CO_2, respectively[1]. The p_i value is influenced by climatic factors like amount of rainfall and light. Slight alteration in these climate governed parameters affects the stomatal conductance, resulting change in p_i values that alter the $\delta^{13}C$ values of C_3 plants. During low rainfall condition plants narrow their stomatal opening to prevent water loss and leads to reduction in p_i value, resulting in an increase in $\delta^{13}C$ values of C_3 plants and vice-versa. The $\delta^{13}C$ of modern C_3 plants and SOM responds principally to rainfall variations[2,3]. Thus, it can be suggested that $\delta^{13}C$ values

of organic matter in sedimentary archives derived from C_3 vegetation will potentially reflect paleoprecipitation[4,5]. Based on this concept, the variability in the Indian Summer Monsoon (ISM) precipitation has been reconstructed for last 12.7 ka. Moreover, the variations in magnetic susceptibility are also found to reflect paleoclimatic changes, because χlf depends on the concentration and mineralogy of magnetic grains. A comparison of χlf with a variety of other geochemical and lithologic indicators (e.g. $\delta^{13}C$, TOC etc.) reveals several defined co-relatable features, which are traditionally used to understand climate change (e.g. high TOC and low magnetic susceptibility).

(2) Geomorphology, geology, vegetation and climate of the study area

The present study has been carried out on a proglacial peat/bog profile (27° 54' 16.28" N; 88° 31' 31.32" E) retrieved from the Chopta Valley, north Sikkim, India. The study site lies in the transition zone between the dry steppe of the Tibetan plateau in the north and the sub-humid Himalayan climate in the south (Fig. 1, 2). Sikkim, a small state with an area of approx. 7299 km^2, is one of the places in the North East Indian Himalaya (NEIH) that has been considered as 'biodiversity hotspots'[6-9]. The region is drained by the Tista River that flows from almost north to south and Chopta Valley lies in its upper reaches. Strategically the state is very important as it shares international borders with countries like Bhutan, China and Nepal Himalaya[10,11]. The region constitutes of the rocks of Lesser, Central and the Tethys Himalaya[12]. The state exhibits a complex topography with sheer altitudinal gradient that influences the vegetation and local weather patterns. These complex characteristics have resulted into existence of microclimatic conditions at small distances and shelter distinctive vegetation and wildlife[13]. The vegetation of the state is represented by the sub-Himalayan wet mixed forests, sub-tropical pine forests, wet temperate forests, mixed coniferous forests, eastern oak-Hemlock forests, Oak-fir forests, moist

alpine scrubs and dry alpine scrubs[14,15]. The study area is extensively covered by grasses and the local communities use these proglacial valleys as a pasture land for grazing their herds. This proglacial valley is formed due to the damming of melt water stream (Chopta Chu) by late Quaternary recessional moraines resulting in the formation of this flat outwash plain that is filled with finer sediments[11].

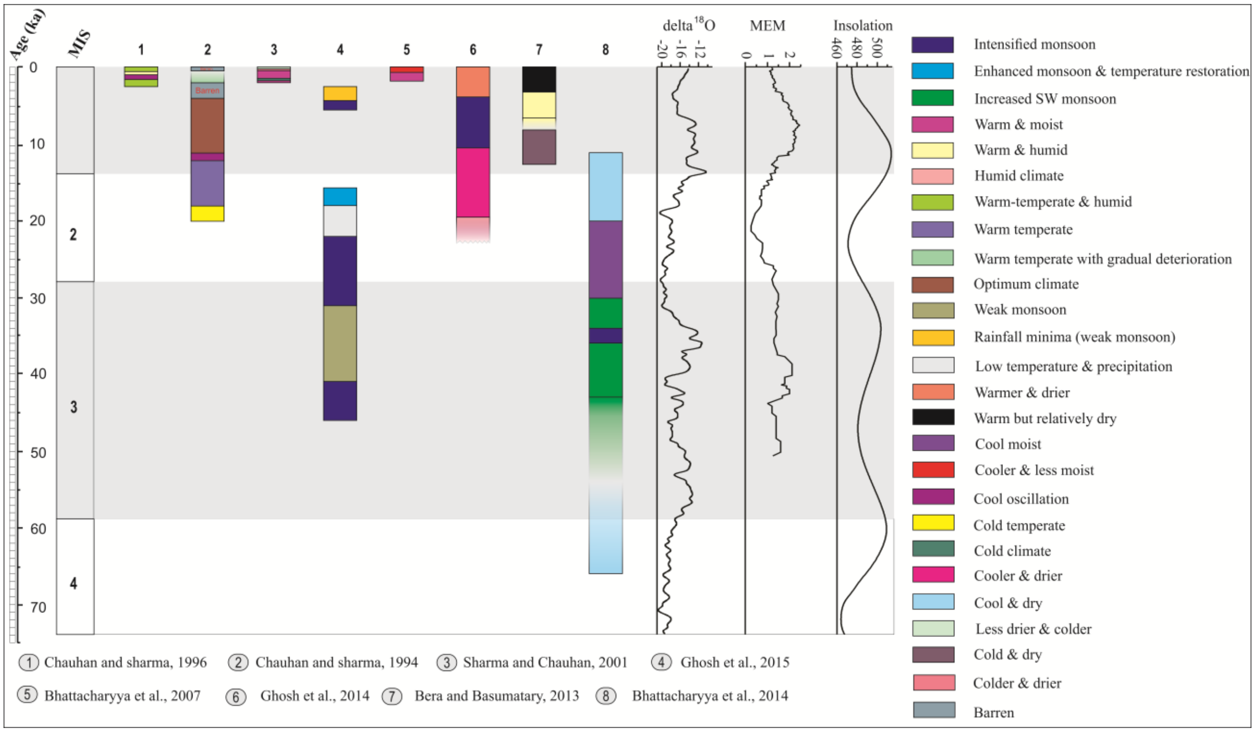

Fig.S1. Comparison of different climatic events established by different workers (1-8) from the north eastern Himalaya, India[37-44] and their correlation with that of the palaeoclimate proxy records via. Oxygen isotopic record of Guliya ice core[45], Effective moisture[46] and Changes in insolation (June insolation 30° N)[47].

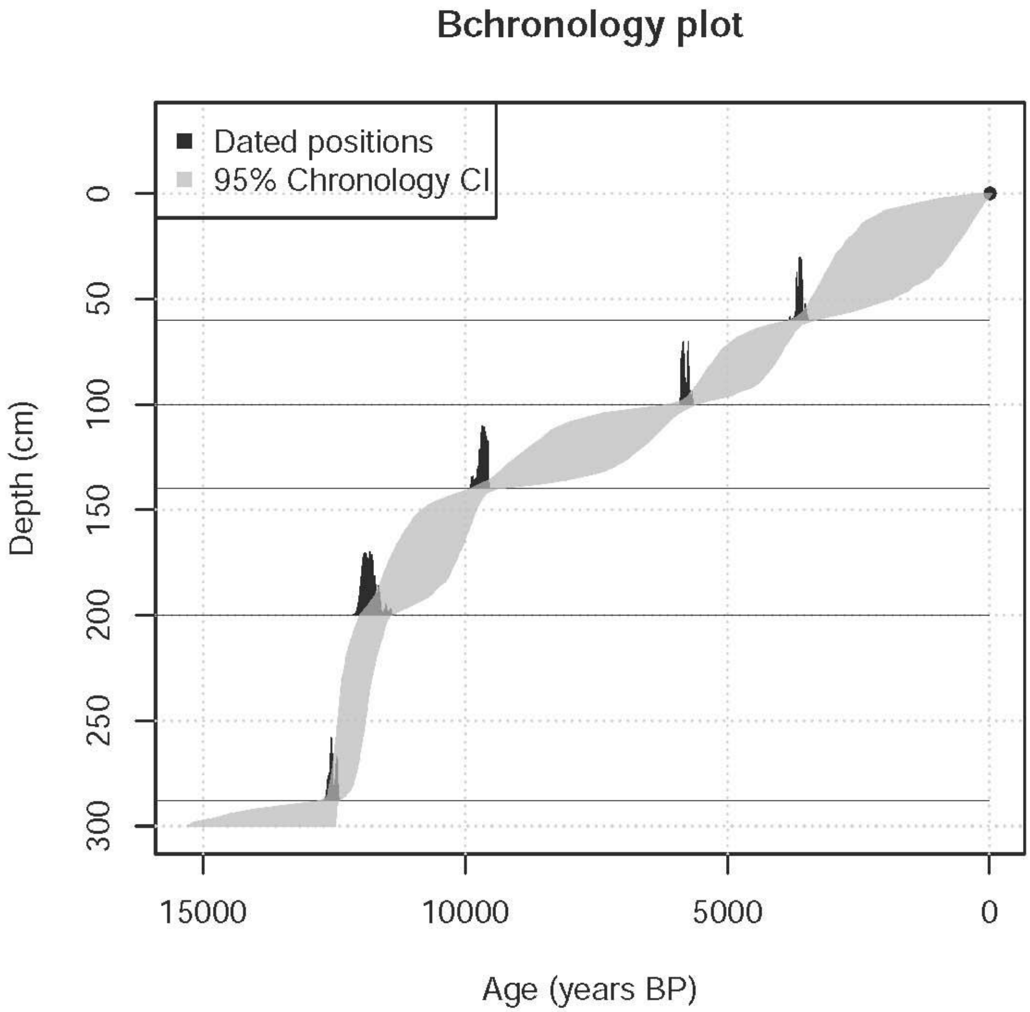

Fig. S2. Basian age depth chronology for the profile.

Climatologically, the state has distinct and wide meteorological variations resulting from complex topography and steep altitudinal gradient. Towards the south (foot hills), the state experiences a subtropical weather while the northern sector (towards Trans Himalaya) experiences tundra type weather. There is a significant and progressive decline in the precipitation gradient from the low altitude southern parts to high altitude northern sector. Since,

the meteorological data is short near to sampling site, therefore, we obtained meaningful climate data from the gridded temperature and precipitation datasets of nearest grid points (22.75N, 87.75 E West Bengal; 27.25 N, 88.75 E Gangtok Sikkim; 028.25 N 88.75E Gurudongmar Sikkim) through Climatic Research Unit (CRU TS.3.24, 0.5 latitude x 0.5 longitude, 1901-2015 CE)[16]. The analysis of CRU TS 3.24 data suggests that the Mean annual temperature (MAT) are around ~ 26.78^{o}C, 12.4^{o}C, -2.45^{o}C while the mean annual precipitation (MAP) are ~1470.7, 2215.9 and 1085.5 mm for West Bengal; Gangtok, Sikkim and Gurudongmar, Sikkim respectively. According to Körner[17], alpine vegetation shows high plant diversity representing nearly 30% of the total flora of Sikkim. The vegetation complex, however, can be broadly classified into three categories: (i) The low altitudinal alpine zone (Shrubland) dominated by phanerophytes and chaemophytes; (ii) The intermediate altitudinal alpine zone represented by the alpine meadows; dominated by geophytes, hemicryptophytes and cryptophytes; and (iii) the high altitudinal alpine zone (trans Himalayan alpine steppe) dominated by hemicryptophytes[11,18].

(3) Materials and Methods, Samples collection

Samples for isotopic ($\delta^{13}C$) analysis were collected from a 3m deep sedimentary profile that has been excavated in the proglaical outwash plain (27° 54' 16.28" N: 88° 31' 31.32" E; ~4000 m asl) of the Chopta valley. Lithologically, the profile has two distinct parts i.e. the top brown to dark brown peat/bog and the bottom light grey fine sandy silty part (Fig. 2). Stratigraphically, from bottom, 10 cm of the profile consists of a silty layer which is overlain by a grey silty-sandy layer of around 85 cm thickness. Sedimentologically and texturally this zone represents a typical succession of glacial sediments deposits by a meltwater stream under favorable melting conditions. A 5 cm dark peaty layer overlies the silty horizon. Brown husky layer with partially decomposed organic material of ~10 cm thickness is present and is overlain by a grey silty layer

of 10 cm thickness and a silty-sandy horizon of 20 cm thickness. The top 160 cm are mostly organic rich peat/bog sediments with brown to dark brown colour (Fig. 2). The upper half of this sedimentary succession is characterized by high organic rich sedimentation and represents a very wet environment with peat/bog formation. The organic material-rich peat is formed by the deposition of layers of sand, silt, clay and plant matter, which undergo microbiological and chemical transformations on a timescale of hundreds of years[19]. In the present study the primary controlling parameter of peat/bog formation could be the changing height of the water table which is directly related to the dynamic fluvial system. The organic rich layers of the profile can be distinguished into three different types on the basis of colour[20]. These are (i) light peat (husky brown layer in the profile) that shows a little decomposition and shows a lot of grass fibers; (ii) dark peat that shows intermediate decomposition and has a dark brown colour with greasy feel and (iii) black peat which is highly decomposed and has a rich dark colour[21]. The bottom most grey sandy/silty sediment layer is clearly a melt water lain sediment layer, highly minerogenous and formed in a redox environment. The growth of peat/bog has taken place once the water level has slightly fallen and environment has changed to oxidizing condition. The husky brown bog layer shows partial decomposition that may be attributed to water level which has created a redox condition. The topmost layer which shows humification in oxidizing conditions produce humic acid and hence the colouring these layers[22].

A total of 150 samples (each representing 2cm of the sediment profile; ~200 gm) were collected from this profile and ~1 gm of the sediment sample was taken after coning and quartering. Sample preparation was done following the procedure discussed in detail in Agrawal et al.[23] and Dubey et al.[11]. The sample were dried at room temperature and subsequently powdered to clay size and poured into 50 ml centrifuge tubes. 5% HCl solution (three times) was added to the

sediments for the removal of carbonates and washed with milli-Q water using a centrifuge machine (~3000 rpm) for the removal of acid and soluble salts. The de-carbonated samples were then dried in a hot air oven with temperature control (< ~45ºC). The oven dried samples were again powdered with an agate mortar to lose any clumps for during the earlier processes. The samples were weighed and carefully packed into tin capsules. The sample filled tin capsules were introduced into the pre-filled and conditioned reactor of Elemental Analyzer (Flash EA 2000 HT) through an auto sampler. The CO_2 gas produced through the combustion was introduced the Continuous Flow Isotope Ratio Mass Spectrometer (CFIRMS, MAT 253) coupled with Con-Flow IV interface for isotopic analysis. In order to check the reproducibility, repeat measurements were done at different intervals. IAEA CH3 was used to calibrate the reference gas and carbon isotopic data has been reported against VPDB. International standards (CH3 and CH6) as well as internal standards (Sulfanilamide) were run to check the accuracy for the CO_2 measurements with an external precision of ±0.1‰ (1σ). Total organic carbon (TOC) was calculated from the peak area obtained from the sum of the integrated m/z 44, 45 and 46 signal measured in the CFIRMS[24]. All samples were analyzed in the Stable Isotope Laboratory, Birbal Sahni Institute of Palaeosciences (BSIP), Lucknow.

Mass specific magnetic susceptibility (χ_{lf}) measurements of the samples were carried out using a Bartington MS2B dual-frequency sensor on a low frequency of 0.465 kHz and x0.1 sensitivity. The samples were packed and weighed in standard 10cc volume plastic vials using a thin foil wrap. Each sample was measured thrice and the results averaged. Air measurements were made between samples to correct for thermally induced drift. All samples were analyzed in Birbal Sahni Institute of Palaeosciences (BSIP), Lucknow.

For establishing the chronology of this sedimentary peat/bog profile six samples have been dated by the AMS ^{14}C radiocarbon dating technique at AMS Radiocarbon Dating Laboratory, Direct-AMS, USA (Table S2). The sample at the depth of 220 cm was exceptionally low considering the stratigraphy, and hence not considered for the age-depth model. Before that all the ages were calibrated using *intcal13* and the calibrated ages (2 σ) are given in Table S2. Assuming the modern sample is of 2±1 a, the age-depth was modeled and this is given in Fig. S2. From the age-depth model, the ages were interpolated for every sample location. The calibration and the age-depth model were done in **R** software[25] using *Bchron* package[26]. The PMIP3 simulated CSIRO Mk3L, IPSL-CM5A-LR and COSMOS-ASO climate model output are used for the spatial extent of ISM (Fig. 4a, b). To get stable efficiency outputs, model was coupled with atmosphere, ocean, sea ice and land surface. The atmospheric CO_2 concentration was stabilized through the preindustrial values (detail of the model can be seen on https://esgf-data.dkrz.de/projects/esgf-dkrz). The calculations are made by CDO version 1.8.2[27] and figures were visualized though GrADS version 2.0.a9[28].

(4) Results

The TOC in the de-carbonated samples of profile is highly variable and range from 0.01 to 67.4% (Table S1). Lower part of the sediments profile (~300 to 202 cm depth), deposited during ~12.7 to11.8 ka, is mainly composed of the light grey fine sandy silt and is characterized by low TOC which range from 0.01 to 1.93. Following this, TOC content increases rapidly from 202 to 186 cm depth (from ~11.8 to 11.3 ka) and reaches up to ~ 47% in the brown husky layer with partially decomposed organic material. Subsequently, TOC content decreases abruptly and relatively low TOC observed in the grey silty layer at ~180 to 170 cm depth (~11.1 to 10.2 ka).

Table S1. Stable carbon isotopic values (‰; VBDB), along with the sample identifier, depth, age, low field magnetic susceptibility, rainfall reconstructed by Kohn's and Basu's models and the average of both the reconstructed rainfall estimates.

Identifier	Depth (cm)	Age (BP)	$\delta^{13}C$ (‰;VPDB)	TOC	Klf ($*10^{-7}$ m^3kg^{-1})	Kohn-PPT (mm/yr)	Basu_PPT (mm/yr)	Average (Kohn/Basu)
	0	0						
CHP 1	2	119	-26.8	15.19	0.473309013	1046	1045	1046
CHP2	4	239	-26.6	38.55	0.52401814	979	1005	992
CHP3	6	360	-27.2	35.47	0.272780289	1221	1140	1180
CHP4	8	480.5	-27.4	36.81	0.021542439	1337	1195	1266
CHP 5	10	603	-27.1	23.39	0.03514395	1194	1126	1160
CHP 6	12	724	-27.1	27.34	0.043862535	1192	1125	1159
CHP7	14	844	-26.8	24.12	0.102251449	1024	1032	1028
CHP8	16	965	-27.6	24.20	0.011849324	1442	1241	1341
CHP9	18	1086	-27.3	27.93	-0.00948047	1286	1171	1229
CHP10	20	1206	-27.5	24.80	0.016724198	1408	1226	1317
CHP11	22	1325	-27.1	29.43	0.02456761	1165	1111	1138
CHP12	24	1446	-26.7	33.46	-0.040836745	1002	1019	1010
CHP13	26	1567	-27.2	34.58	0.042606429	1216	1137	1176
CHP 14	28	1687	-26.7	43.71	0.036277625	993	1013	1003
CHP15	30	1809	-26.7	50.13	-0.065929804	992	1013	1003
CHP16	32	1928.5	-26.7	53.26	0.061244957	993	1014	1004
CHP 17	34	2049	-26.9	67.36	0.072389455	1071	1060	1066
CHP18	36	2169	-27.2	31.63	0.14878568	1214	1136	1175
CHP19	38	2291	-26.8	27.22	0.074407926	1053	1049	1051
CHP 20	40	2412	-26.8	30.00	-0.048029675	1060	1053	1057
CHP 21	42	2531	-26.7	41.50	-0.089612305	1002	1019	1011
CHP22	44	2653	-27.0	20.38	-0.09576526	1113	1083	1098
CHP 23	46	2774	-27.3	22.75	-0.084882438	1288	1172	1230
CHP24	48	2893	-26.9	41.37	-0.0232762	1088	1069	1079
CHP25	50	3011	-26.6	46.13	0.038330037	976	1003	989
CHP26	52	3133	-26.5	32.56	-0.095256239	909	960	935
CHP 27	54	3253	-26.4	21.12	-0.026861863	882	941	912
CHP 28	56	3373	-26.4	-	-0.053401687	898	952	925
CHP29	58	3495.5	-26.5	49.83	-0.091459293	914	963	939
CHP30	60	3615.5	-26.5	49.27	0.078196058	927	971	949
CHP31	62	3727	-26.4	51.80	0.050755872	876	937	907
CHP 32	64	3837	-26.1	55.79	-0.153229982	790	874	832
CHP33	66	3948.5	-26.4	59.51	-0.088504526	898	952	925
CHP34	68	4058.5	-26.5	41.70	0.201878275	922	969	946
CHP35	70	4167	-26.5	50.64	0.091819316	933	976	955
CHP36	72	4278	-26.3	59.16	0.050209121	834	907	871

CHP 37	74	4386	-26.0	59.55	0.166992572	743	837	790
CHP 38	76	4497	-26.3	29.10	-0.050168837	842	913	877
CHP 39	78	4606.5	-26.4	31.82	-0.050607681	870	933	902
CHP 40	80	4716	-25.6	46.80	-0.043633444	644	750	697
CHP41	82	4826.5	-26.2	46.73	-0.090027909	832	906	869
CHP 42	84	4936	-26.0	45.40	-0.062237287	752	844	798
CHP43	86	5044.5	-26.4	13.83	-0.060254273	875	936	906
CHP44	88	5155	-26.4	22.10	-0.036081172	891	948	920
CHP 45	90	5265	-26.4	16.20	0.053022525	880	940	910
CHP46	92	5373	-26.1	33.53	-0.041002091	792	876	834
CHP47	94	5481	-26.0	32.35	-0.11119491	746	840	793
CHP48	96	5588	-26.4	27.75	0.204133929	894	950	922
CHP 49	98	5697.5	-26.4	-	0.204680089	888	945	917
CHP 50	100	5823	-26.4	26.18	0.231967477	882	941	911
CHP51	102	5999.5	-26.3	41.59	-0.060809325	865	929	897
CHP52	104	6193	-26.6	-	0.025580027	963	995	979
CHP53	106	6383	-26.9	31.08	0.032140463	1072	1060	1066
CHP 54	108	6578.5	-26.8	29.08	0.06983545	1024	1033	1029
CHP 55	110	6770	-26.7	-	0.040691208	1013	1025	1019
CHP 56	112	6967	-26.7	31.43	0.027115388	1001	1018	1010
CHP57	114	7160	-26.8	40.96	0.031801305	1025	1033	1029
CHP58	116	7351	-26.4	30.29	-0.045101117	879	939	909
CHP59	118	7544	-26.4	10.94	0.013748256	902	955	929
CHP60	120	7736.5	-26.6	12.78	0.028549288	971	1000	985
CHP61	122	7934	-26.8	8.40	-0.07316627	1029	1035	1032
CHP62	124	8128	-26.9	-	0.026031789	1105	1079	1092
CHP63	126	8321	-27.1	8.90	0.096617557	1187	1122	1154
CHP64	128	8517	-27.3	28.32	0.492607216	1303	1179	1241
CHP65	130	8709	-27.2	37.39	0.170468434	1226	1142	1184
CHP66	132	8901	-27.2	29.80	0.195411357	1239	1148	1193
CHP67	134	9092	-27.3	36.23	0.20993638	1303	1179	1241
CHP68	136	9287	-27.4	4.35	0.465158574	1340	1196	1268
CHP69	138	9475	-27.2	2.38	0.029357906	1248	1153	1200
CHP 70	140	9638	-27.1	1.28	0.129128308	1197	1127	1162
CHP71	142	9728	-27.5	6.91	0.299652403	1371	1210	1291
CHP 72	144	9802	-27.4	-	0.131358803	1337	1195	1266
CHP73	146	9872	-27.3	1.62	1.065509187	1304	1180	1242
CHP74	148	9942	-26.6	1.03	1.033260293	959	992	976
CHP75	150	10011	-26.7	0.89	0.933441325	985	1009	997
CHP76	152	10081	-27.3	0.93	1.037217067	1289	1172	1230
CHP77	154	10153	-27.3	0.90	0.872707718	1274	1165	1220
CHP78	156	10224.5	-27.0	1.67	0.559173063	1152	1104	1128
CHP79	158	10296	-26.6	0.70	0.725112988	966	997	982

CHP80	160	10366	-26.4	0.53	0.601295097	899	953	926
CHP81	162	10438	-26.5	0.56	0.549392359	932	975	954
CHP82	164	10511	-27.0	1.17	0.362679459	1132	1094	1113
CHP83	166	10582.5	-27.3	2.09	0.226215416	1295	1175	1235
CHP84	168	10654.5	-26.3	2.81	0.640779187	859	925	892
CHP85	170	10727	-26.1	0.91	0.899634664	796	879	838
CHP86	172	10797.5	-26.4	18.64	1.016955596	876	937	907
CHP87	174	10868	-26.6	-	0.752898246	955	990	973
CHP88	176	10940	-26.8	19.50	0.335800534	1041	1042	1042
CHP89	178	11009	-26.4	-	0.389227251	886	944	915
CHP90	180	11080.5	-26.0	17.57	0.664926726	754	846	800
CHP91	182	11148.5	-26.5	39.06	1.013386469	936	978	957
CHP92	184	11220	-26.2	46.22	1.232696777	827	902	864
CHP93	186	11291.5	-26.3	47.15	1.295080391	850	919	884
CHP 94	188	11358.5	-26.2	42.78	1.026719143	830	904	867
CHP95	190	11427.5	-24.3	20.26	0.41116209	376	422	399
CHP 96	192	11498	-24.8	0.90	0.242435997	452	534	493
CHP 97	194	11567	-24.8	0.42	0.170854744	467	554	511
CHP 98	196	11637.5	-25.0	0.89	0.486500613	492	586	539
CHP 99	198	11711	-25.8	1.61	0.12619242	696	797	747
CHP100	200	11779	-26.8	1.67	0.940309413	1065	1056	1060
CHP101	202	11822	-26.3	1.84	0.719144133	845	915	880
CHP102	204	11843	-26.4	0.32	0.861290928	890	947	918
CHP103	206	11861	-26.4	0.19	1.290319083	893	949	921
CHP104	208	11877.5	-26.9	0.26	1.134160527	1077	1063	1070
CHP105	210	11896.5	-26.5	0.31	-	936	978	957
CHP 106	212	11914	-26.4	0.98	0.484835637	882	942	912
CHP107	214	11930	-26.6	0.36	0.214776632	957	991	974
CHP108	216	11945	-26.9	0.37	0.484647576	1110	1081	1095
CHP109	218	11960.5	-27.0	1.76	0.669540474	1118	1086	1102
CHP110	220	11975.5	-26.9	0.61	0.718167138	1066	1057	1062
CHP111	222	11991	-25.8	0.83	0.761458176	692	794	743
CHP112	224	12006	-26.8	1.72	0.804749214	1024	1032	1028
CHP113	226	12022	-26.8	1.89	0.906174968	1024	1032	1028
CHP114	228	12038	-26.4	-	1.022909488	893	949	921
CHP115	230	12053	-26.1	0.55	1.139644008	779	866	822
CHP 116	232	12069	-26.6	0.15	0.99676533	965	996	980
CHP117	234	12085	-26.0	0.23	0.853886652	740	835	788
CHP118	236	12101	-26.8	0.29	0.849213628	1032	1037	1035
CHP119	238	12117.5	-26.7	0.23	0.743188945	1019	1029	1024
CHP120	240	12133	-26.7	0.84	0.467811004	989	1011	1000
CHP121	242	12149	-27.0	0.34	0.591753397	1154	1105	1129
CHP 122	244	12165	-27.0	0.06	0.449328626	1117	1085	1101

CHP123	246	12181.5	-26.1	0.13	0.803430155	799	881	840
CHP124	248	12198	-26.3	0.31	0.555390762	867	931	899
CHP 125	250	12216	-26.6	1.93	0.482063482	951	987	969
CHP126	252	12233	-26.8	1.76	0.479914999	1060	1053	1056
CHP127	254	12251	-26.5	-	0.665798415	933	976	954
CHP128	256	12268.5	-26.2	0.78	1.06753188	822	898	860
CHP129	258	12286	-26.5	0.77	1.173636238	935	977	956
CHP130	260	12302	-26.4	0.41	0.896024352	895	950	923
CHP 131	262	12318	-26.7	0.19	0.70141428	1009	1023	1016
CHP132	264	12334.5	-27.0	0.15	0.540205596	1144	1100	1122
CHP133	266	12352	-27.9	0.04	0.598559641	1654	1324	1489
CHP134	268	12369	-27.9	0.01	0.423076272	1650	1323	1486
CHP135	270	12386.5	-28.0	0.05	0.39649057	1696	1340	1518
CHP 136	272	12402.5	-27.8	0.06	0.412359644	1587	1299	1443
CHP 137	274	12420	-27.7	-	0.420395108	1527	1276	1401
CHP 138	276	12437	-27.6	0.06	0.428430571	1469	1252	1361
CHP 139	278	12452.5	-26.2	0.99	0.603111587	813	892	853
CHP 140	280	12471	-25.9	-	0.416067041	731	827	779
CHP141	282	12488	-25.7	0.25	0.639865582	657	762	710
CHP142	284	12506	-26.5	0.73	0.863664124	941	981	961
CHP143	286	12523	-26.5	-	0.90384688	933	975	954
CHP144	288	12549.5	-26.5	2.59	1.119178629	925	970	948
CHP145	290	12587	-26.6	-	1.208713913	944	983	964
CHP146	292	12621	-26.6	0.47	0.940694529	964	995	980
CHP147	294	12644	-27.1	0.40	0.574944836	1168	1113	1140
CHP148	296	12668.5	-27.2	0.36	0.872463809	1208	1133	1170
CHP 149	298	12693	-26.2	0.35	0.938628867	805	886	845

Further, TOC content show decreasing trend and low TOC content (~0.59 to 6.91%) is observed in a silty-sandy horizon at ~170 and 136 cm depth (~10.7 to 9.3). Post 130 cm depth show highly variable TOC range from 8 to 67% and highest TOC content (67%) observed at ~34 cm (~2 ka) in the black peat layer (Fig. 3). The $\delta^{13}C$ values in lower half of the sediment profile are also highly variable. This section of the profile is deposited during ~12.7 and 10.6 ka (between depth ~300 and 166 cm) and is characterized by alternate layers of silt and sand punctuated with dark and black peat layers. Here, we have observed some abrupt changes in $\delta^{13}C$ values ranging

between –28.0 and –24.3‰, and the highest fluctuations recorded in the entire profile. An abrupt negative excursion in the $\delta^{13}C$ values (–28.0‰) is recorded between 12.4 and 12.3 ka between ~276 and 264 cm depth. While the most positive excursion in the $\delta^{13}C$ values (–24.3‰) is noticed between 11.7 and 11.4 ka between ~198 and 188 cm depth. This transient phase is followed by a gradual decrease in the $\delta^{13}C$ values during ~10.6 and 8 ka (between depths 166 to 124cm mostly comprised of black peat). Lithologically, this phase is overlain by dark peat that extends between ~124 and 50 cm (deposited between ~8 and 3ka). This zone is characterized by a relatively higher isotopic value that reached up to –25.6‰ at ~80cm depth. The upper 50 cm (deposited between ~3 ka and present), sediment is comprised of dark peat and interspersed by a black peat layer (from ~40 to 20cm depth) showing isotopically fluctuating trend varying from –27.6 to –26.6‰. It is interesting to note that in past 300 yr, the isotopic values show a stabilizing trend (–26.6‰) similar to contemporary values (Fig. 3). The magnetic susceptibility (MS) show a definite fluctuating trend throughout the sedimentary profile and significantly reflects the lithology changes. The sandy horizons show maximum MS values while the organic peaty layers are characterized by minimum MS values. The MS values in the lower half of the sedimentary profile that is characterized by coarser sediments (high sedimentation rate) show highly variable values. It is observed that during ~12.7 to ~10 ka (300 to 150 cm) show abrupt significant fluctuations in MS ranging from 0.13 to 1.3 ($*10^{-7}m^3kg^{-1}$). It has to be noted that a broad positive correlation is observed between MS and mean rainfall whereas an anti-correlation is observed with TOC and $\delta^{13}C$ values. The upper half of the profile is dominated by organic reach peaty sediments and shows lower MS values ranging between -0.15 and 0.52 ($*10^{-7}m^3kg^{-1}$). There is a prominent anti-correlative trend is observed between MS and $\delta^{13}C$ values, although

the MS values are significantly lower that is attributed to the peaty nature of the sediments. However, our interpretation of MS is in good agreement with carbon isotopic data (Fig. 3).

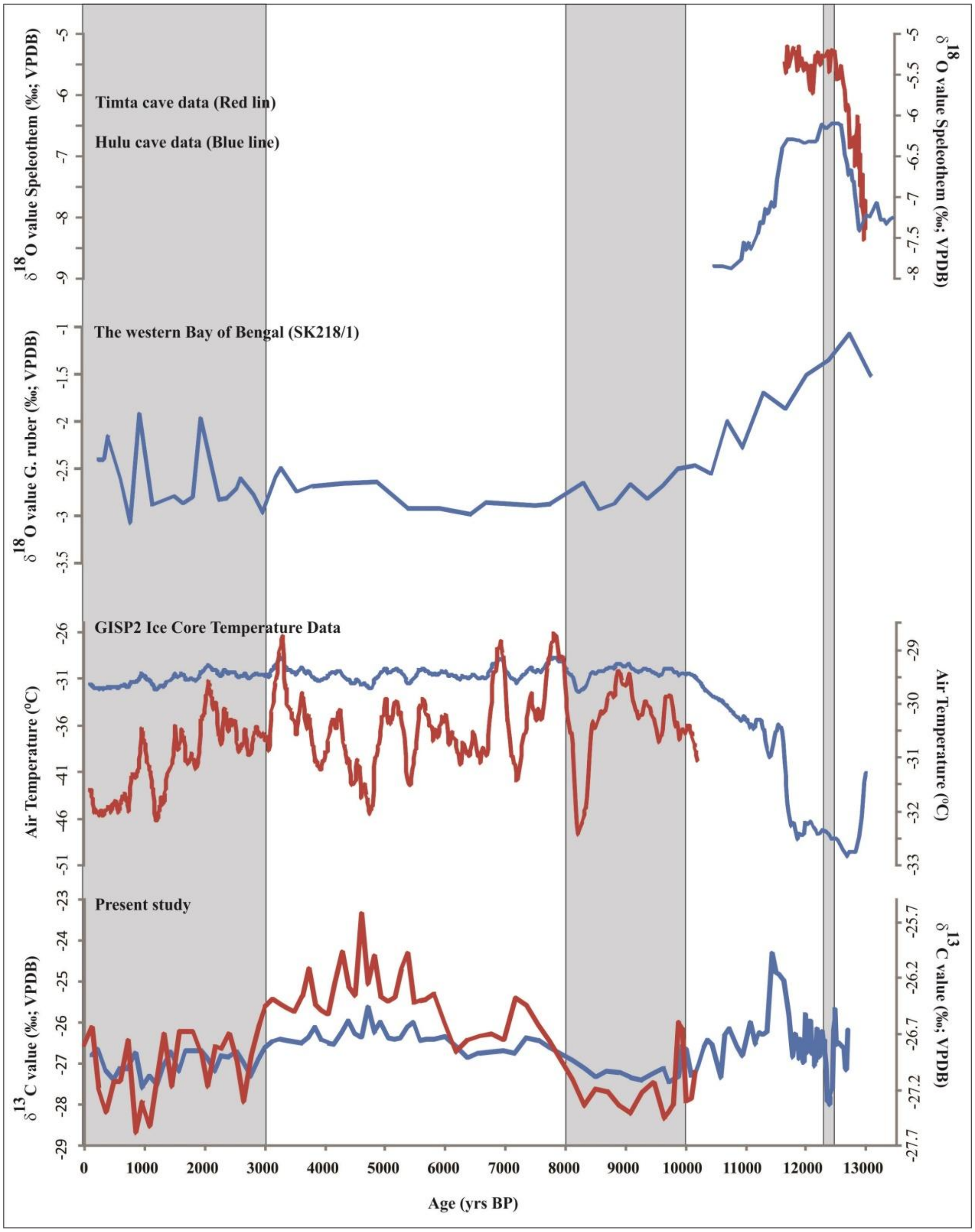

Fig. S3. Correlation of reconstructed rainfall with other regional proxies via. GISP2 ice core temperature data, Core SK 218/1 from the western way of Bengal, Hulu and Timta cave data.

(5) $\delta^{13}C$ values of organic matter as palaeo-precipitation indicator

The carbon isotope ratio of C_3 plants and modern surface sediments (C_3 vegetation dominated) is inversely correlated with the rainfall amount[2,3,29-35]. Kohn[35] and Diefendorf et al.[34] have used the global data of $\delta^{13}C$ values of C_3 plants to demonstrate the dependency of $\delta^{13}C$ on the mean annual rainfall at a particular location. A recent study from the Gangetic plains, India also suggested an increases of $\delta^{13}C$ values with a decrease in rainfall amount. Rao et al.[3] compiled 10,000 $\delta^{13}C$ values of modern plants and surface soils globally and showed that $\delta^{13}C$ values of modern C_3 plants and surface soils under pure C_3 vegetation are significantly negatively correlated with MAP. All these studies confirm that $\delta^{13}C$ of organic matter sourced from C_3 vegetation can be used for paleoprecipitation reconstructions. In this series, quantitative palaeoprecipitation reconstruction based on $\delta^{13}C$ values has been applied to study Europian loess[4] and the loess of the western China Plateau[5]. These studies have demonstrated the $\delta^{13}C$ values of any terrestrial ecosystem dominated or composed entirely of C_3 plants have a potential to be used for paleoprecipitation reconstructions. In the present study, we have used 100yr average CRU data of precipitation and cross checked our modern $\delta^{13}C$ based precipitation values with it. The correlations are in great agreement and hence give a confidence in the palaeoprecipitation reconstructions. For this we have used the equations given by Kohn[35] and Basu et al.[2]. Kohn[35] has used $\delta^{13}C$ values to calculate modern monsoonal rainfall and suggested that the $\delta^{13}C$ values of modern C_3 plants primarily depends on MAP and to some extent on altitude and latitude. Therefore, in the present study the following equation of Kohn[35] has been used

$\delta^{13}C$ (‰, VPDB) $=-10.29 + 1.90 \times 10-4$ Altitude (m) $-$ 5.61log10 (MAP + 300 mm/year) $-$ 0.0124 Abs (latitude°).

In addition, the rainfall calculation based on the Kohn[35] equation are cross checked by the relationship given by Basu et al.[2] from the Ganga Plain, India. According to Basu et al.[2] the $\delta^{13}C$ values of modern C_3 plants are increases by 0.4‰ with a decrease in rainfall amount by 100 mm and vise verse.

(6) Model outputs for paleoprecipitation extent

From Last Glacial Maximum (LGM) to Historical period model shows nearly similar pattern to the sedimentary proxy data of Kohn and Basu. During mid Holocene period model shows a continuous negative anomaly of the precipitation over India region. That is the period of Indus valley civilizations ups and downs (3.5k to 6k) (Fig. 4a)[36]. In historical period, during preindustrial the precipitation anomaly is positive over the Indian region. However, it decreased in-between and then again it reconstructed to the normal but the extreme precipitation anomaly (positive as well as negative) years are increased (Fig. 4a). The annual and JJAS (June, July, August, September) precipitation climatology is shown in figure 4b, to see ISM extent from Holocene to historical period. That clearly justifies the diverse precipitation from LGM to historical (Fig. 4b).

Table S2. Details of the AMS ^{14}C dates obtained on five samples at different depths of the peat/bog sedimentary profile, Chopta valley, north Sikkim.

S. No.	Sample ID	Depth (cm)	Lab. code	Age BP	Error (1□)	Calibrated age range (a) CI (2 sigma)	
1	CH-P-30	60	D-AMS 016224	3372	42	3548	3700
2	CH-P-50	100	D-AMS 016225	5035	30	5711	5895
3	CH-P-70	140	D-AMS 016226	8713	45	9548	9795
4	CH-P-100	200	D-AMS 016227	10163	60	11604	12061
6	CH-P-144	288	D-AMS 016229	10560	45	12421	12638

References

1. Farquhar, G. D., Ehleringer, J. R. & Hubick, K. T. Carbon isotope discrimination and photosynthesis. *Annu Rev Plant Physiol Plant Mol Biol* 40, 503–537 (1989).
2. Basu, S., Agrawal, S., Sanyal, P., Mahato, P., Kumar, S. & Sarkar, A. Carbon isotopic ratio of modern C_3–C_4 plants from the Gangetic Plain, India and its implications to paleovegetational reconstruction. *Palaeogeography, Palaeoclimatology, Palaeoecology,* (2015).
3. Rao, Z., Guo, W., Cao, J., Shi, F., Jiang, H. & Li, C. Relationship between the stable carbon isotopic composition of modern plants and surface soils and climate: a global review. *Earth Sci. Rev.* 165, 110-119 (2017)..
4. Hatté, C. *et al*. $\delta^{13}C$ of loess organic matter as a potential proxy for paleoprecipitation. *Quat. Res.* 55, 33–38 (2001).
5. Rao, Z. G., Chen, F. H., Cheng, H., Liu, W. G., Wang, G. A., Lai, Z. P. & Bloemendal, J. High resolution summer precipitation variations in the western Chinese Loess Plateau during the last glacial. *Sci. Rep.* 3, 2785 (2013).
6. Rao, R. R. Vegetation phytogeography of Assam–Burma. In: Mani MS (ed.) *Ecology Biogeography of India*. The Hague: Dr. W. Junk B.V. Publishers, 204–246 (1974).
7. Hajra PK, Verma DM and Giri GS (eds) (1996) *Materials for the Flora of Arunachal Pradesh*, vol. I. Calcutta: Botanical Survey of India.
8. Singh, P. & Chauhan, A. S. Plant diversity in Sikkim Himalaya. In: Hajra PK and Mudgal V (eds) *Plant Diversity Hotspots of India: An Overview*. Kolkata: Botanical Survey of India, pp. 137–158 (1997).
9. Brooks, T. M. et al. Global biodiversity conservation priorities. *Science* 313, 58–61 (2006).

10. Sikkim SAPCC (2011) Sikkim Action Plan on Climate Change (2012–2030). Available at: http://www.moef.nic.in/sites/default/files/sapcc/Sikkim.pdf.

11. Dubey, D., Ghosh, R., Agrawal, S., Quamar, MF., Morthekai, P., Sharma, R. K., Sharma, A., Pandey, P., Srivastava, V., & Ali, S. N. Characteristics of modern biotic data and their relationship to vegetation of the Alpine zone of Chopta valley, North Sikkim, India: Implications for palaeovegetation Reconstruction. *Holocene* (2017).

12. Schaller, G. B. *Mountain Monarchs: Wild Goat Sheep of the Himalaya*. Chicago, IL: University of Chicago Press (1977).

13. Chettri, N., Sharma, E., & Deb, D. C. Bird community structure along a trekking corridor of Sikkim Himalaya: A conservation perspective. *Biological Conservation* 102, 1–16 (2001).

14. Champion, H. G. & Seth, S. K. *A Revised Survey of Forest Types of India*. New Delhi: Government of India Press, 404 (1968).

15. Mehra, M. S., Pathak, P. C. & Singh, J. S. Nutrient movement in litter fall and precipitation components for Central Himalayan forests. *Ann. Bot.* 55, 153-170 (1985).

16. Harris, Jones, P. D., Osborn, T. J. & Lister D. H. Updated high-resolution grids of monthly climatic observations – the CRU TS 3.10. *Int. J. Climatol*. 34, 623-642 (2014).

17. Körner, C. Alpine Plant Life: Functional Plant Ecology of High Mountain Ecosystems. Berlin: Springer-Verlag, 343 (1999).

18. Telwala, Y. Climate change alpine flora in Sikkim Himalaya. In: Arrawatia, ML, Tambe, S (eds) Climate Change in Sikkim-Patterns, Impacts and Initiatives. Sikkim, India: Information and Public Relations Department, Government of Sikkim, 103–124 (2012).

19. Stach, E., Mackowsky, M.-Th., Teichmuller, M., Taylor, G. H., Chandra, D., Teichmuller, R., Stach's Textbook of Coal Petrology. Borntraeger, 2nd Ed., Berlin (1975).
20. IPS, International Peat Society, Jyväskylä, Finland, http://www.peatsociety.org/ (2013).
21. Saito, B. & Seckler, M. M. Alkaline extraction of humic substances from peat applied to organicmineral fertilizer production. *Brazilian Journal of Chemical Engineering* 31, 675–682 (2014).
22. Xintu, L. Conditions of peat formation. *Encyclopedia of Life Support Systems* (EOLSS), Vol. II, 11 (2008).
23. Agrawal, S. *et al.* Stable ($\delta^{13}C$ and $\delta^{15}N$) isotopes and magnetic susceptibility record of late Holocene climate change from a lake profile of the northeast Himalaya. *J. Geol. Soc. India*, 86, 696-705 (2015).
24. Jensen, E. S. Evaluation of automated analysis of ^{15}N and total N in plant material and soil. *Plant and Soil* 133, 83-92 (1991).
25. R Core Team. R: A Language and Environment for Statistical Computing. R Foundation for Statistical Computing, Vienna, Austria. URL https://www.R-project.org/ (2017).
26. Parnell, A. Bchron: Radiocarbon dating, age-depth modelling, relative sea level rate estimation, and non-parametric phase modelling. R package version 4.1.1; 2015. Available: http://CRAN.R-project.org/package=Bchron. Accessed 3 January 2016.
29. Stewart, G. R., Turnbull, M. H., Schmidt, S. & Erskine, P. D. ^{13}C natural abundance in plant communities along a rainfall gradient: a biological integrator of water availability. *Aust. J. Plant Physiol*. 22, 51–55 (1995).
30. Wang, G. A., Han, J. M. & Liu, T. S. The carbon isotope composition of C3 herbaceous plants in loess area of northern China. *Sci. China Ser.* 46, 1069–1076 (2003).

31. Swap, R. J., Aranibar, J. N., Dowty, P. R., Gilhooly, W. P. Macko, S. A. Natural abundance of C-13 and N-15 in C-3 and C-4 vegetation of southern Africa: patterns and implications. *Global Change Biol.* 10, 350–358 (2004).

32. Liu, W. G., Feng, X. H., Ning, Y. F., Zhang, Q. L, Cao, Y. N. & An, Z. S. delta C-13 variation of C-3 and C-4 plants across an Asian monsoon rainfall gradient in arid northwestern China. *Global Change Biol*. 11, 1094–1100 (2005).

33. Zheng, S. X. & Shangguan, Z. P. Spatial patterns of foliar stable carbon isotope compositions of C3 plant species in the Loess Plateau of China. *Ecol. Res.* 22, 342–353 (2007).

34. Diefendorf, A. F., Mueller, K. E., Wing, S. L., Koch, P. L. & Freeman, K. H. Global patterns in leaf 13C discrimination and implications for studies of past and future climate. *Proc. Natl. Acad. Sci. U. S. A.* 107, 5738–5743 (2010).

35. Kohn, M. J. Carbon isotope compositions of terrestrial C3 plants as indicators of (paleo) ecology and (paleo) climate. *Proc. Natl. Acad. Sci. U. S. A.* 107, 19691–19695 (2010).

36. Dixit, Y., Hodell, D. A. & Petrie, C. A. (2014). Abrupt weakening of the summer monsoon in northwest India ~ 4100 yr ago. *Geology* 42, 339–342.
